# Attitudes of health care providers regarding female genital mutilation and its medicalization in Guinea

**DOI:** 10.1371/journal.pone.0249998

**Published:** 2021-05-13

**Authors:** Mamadou Dioulde Balde, Sarah O’Neill, Alpha Oumar Sall, Mamadou Bailo Balde, Anne Marie Soumah, BoubacarAlpha Diallo, Christina Catherine Pallitto

**Affiliations:** 1 Cellule de Recherche en Sante de la Reproduction en Guinée (CERREGUI), Conakry, Guinea; 2 Ecole de Santé Publique and Faculté de Philosophie et de Sciences Sociales, Université Libre de Bruxelles, Brussels, Belgium; 3 UNDP-UNFPA-UNICEF-WHO-World Bank Special Programme of Research, Development and Research Training in Human Reproduction (HRP), Department of Sexual and Reproductive Health and Research, World Health Organization, Geneva, Switzerland; Guttmacher Institute, UNITED STATES

## Abstract

**Background:**

Guinea has a high prevalence of female genital mutilation (FGM) (95%) and it is a major concern affecting the health and the welfare of women and girls. Population-based surveys suggest that health care providers are implicated in carrying out the practice (medicalization). To understand the attitudes of health care providers related to FGM and its medicalization as well as the potential role of the health sector in addressing this practice, a study was conducted in Guinea to inform the development of an intervention for the health sector to prevent and respond to this harmful practice.

**Methodology:**

Formative research was conducted using a mixed-methods approach, including qualitative in-depth interviews with health care providers and other key informants as well as questionnaires with 150 health care providers. Data collection was carried out in the provinces of Faranah and Labé and in the capital, Conakry.

**Results:**

The majority of health care providers participating in this study were opposed to FGM and its medicalization. Survey data showed that 94% believed that it was a serious problem; 89% felt that it violated the rights of girls and women and 81% supported criminalization. However, within the health sector, there is no enforcement or accountability to the national law banning the practice. Despite opposition to the practice, many (38%) felt that FGM limited promiscuity and 7% believed that it was a good practice.

**Conclusion:**

Health care providers could have an important role in communicating with patients and passing on prevention messages that can contribute to the abandonment of the practice. Understanding their beliefs is a key step in developing these approaches.

## Introduction

Female genital mutilation (FGM) is a harmful practice affecting approximately 200 million women and girls globally and involves the partial or total removal of external female genitalia or other injury to female genital organs for non-medical reasons [1]. Health risks can include immediate risks of hemorrhage and shock, as well as long-term reproductive, obstetric, mental, and sexual health effects [1]. In many places where FGM is performed it is a social norm and endorsed as a traditional, ritual or religious practice, although FGM is not formally prescribed by any religion. Commonly mentioned reasons for practising are control of female sexuality—such as to ensure virginity before marriage and marital fidelity [2–5]; conceptions of purity and aesthetics [2, 5, 6]; or as a way to mark coming of age and status change within the community [7–9]. Guinea has a high prevalence of FGM, as 95% of women have undergone this practice, with 65% occuring between the ages of five to fourteen years with a peak between the ages of five to nine years (37%) [10]. According to the Demographic Health Survery 2018, 58% of women have undergone Type I or II FGM, 10% reported infibulation (type III) and 11% Type IV [10]. Recent figures show that 72% of women between the ages of 15–49 were cut by traditional excisers. Mothers of girls in the 0–14 year age group reported that 59% of those cut were cut by traditional cutters and 35% were cut by a health care provider. The rate of medicalization is twice as high among this age group as compared to adult women, implying an increasing trend in medicalized FGM [10].

The inclusion of the abandonment of FGM as a target within the Sustainable Development Goals shows the international commitment of United Nations Member States not only in ending this harmful practice but also in measuring the progress towards this goal. Population-based surveys indicate that progress towards abandonment needs to be accelerated in Guinea and elsewhere and that health care providers are increasingly asked to carry out FGM on girls [11]. This study constitutes Phase 1 of a formative research project (entitled “Formative research on medicalization and clinical management of FGM in Guinea”) aimed at understanding the attitudes of health care providers regarding FGM, why some health care providers perform the practice, and how the health sector can play a stronger role in abandonment efforts. The findings of the research informed the development of an intervention to promote abandonment of FGM within the health sector in Guinea and other settings with high FGM prevalence [12]. The intervention is being tested in a randomized cluster trial in three countries (Guinea, Somalialand, Kenya) in Phase 2 of the research.

### Study setting and population

Guinea is located on the West African coast and has a population of 11,663,627 inhabitants. Since the 1990s various strategies have been put in place at national and regional level to stop FGM. A ten-year strategic plan (2003–2013) was also drawn up and implemented in accordance with the recommendations of the International Conference held in Addis Ababa (Ethiopia) in February 2003 on the theme "Zero Tolerance to FGM". The law prohibiting the practice was passed in 2008 (articles 407 to 409 of the ‘code de l’Enfant Guinéen’) according to which FGM is punishable with fines of up to 3 million gnf and up to 3 years emprisonment. A National Committee to Combat FGM was established in 2011 under the supervision of the Ministry of Social Welfare, Promotion of Women and Children. A joint decree of the Ministries of the Interior, Social Affairs and Health penalising the practice of excision in Guinea was promulgated in 2010. Depite these provisions no significant decrease FGM has been observed. The health sector can play an important role in a multi-sectoral response to the abandonment of FGM, yet until 2018 there had not been a systematic approach to strengthen the health sector to address FGM in Guinea. In 2018, a national action plan for the health sector was developed to plan and implement activities to improve the treatment and care of girls and women affected by FGM and to prevent the practice. The research findings of this study informed the development of the action plan. This study identified the gaps, the opportunities, and the possibilities for building a more comprehensive response to FGM by better understanding how health care providers, health programme planners, patients and community members view the role of the health sector in this regard, and how they themselves view the practice.

### Methods

This formative research employed a mixed methods approach, including qualitative and quantitative methods. Participants for the qualitative part of the study were selected from three areas of the country—Faranah, Labé and Conakry to reflect geographic and ethnic representation as well as urban and rural perspectives. A total of 20 in-depth interviews were conducted with health care providers, including 8 with midwives (outside Conakry, 4 in urban and 4 in rural areas), 8 with nurses (outside Conakry, 4 in urban and 4 in rural areas) and 4 with medical doctors (2 doctors in Faranah and 2 doctors in Labé). Also, 13 interviews were conducted with key-informants (health system managers, heads of schools of nursing and midwifery and the faculty of Medicine, representatives of professional associations, and patients finishing consultations). In-depth interviews with health care providers took place at the health centres where they worked.

In Guinea, midwives and nurses all have a training period of 3 years, with the first year as a common core for both options. Specialization (midwives/nurses) takes place in the second and third years with modules specific to the options. For example, obstetric care courses are taught only to future midwives. For the selection of health care providers, the research team facilitated contact with the head facility administrator in each of the selected facilities for a listing of health care providers at the selected sites. Based on the number of providers at the site, and the desired number of respondents per site indicated, a random selection was made to identify potential participants for the quantitative component. For the in-depth interviews, a random sampling was undertaken to select providers according to professional training. Based on the listing of providers, a random selection was conducted in each facility with the "Random plus" software to select one or two providers per site, which ensured an adequate representation of different cadres of providers.

In-depth interviews with patients and focus group discussions among community members were also conducted. In-depth interviews with patients were conducted following their consultation at the health facility. The language of the interview was either French, Soussou, Malinké or Pulaar. The focus group discussions with community members took place in the village meeting places in Faranah (urban: Faranah; rural: Marella), Labe (urban: Labe; rural: Sannoun) and Conakry and were conducted in Soussou, Malinké or Pulaar with similar groups in terms of age, ethnic group, and socio-economic status. Household listings provided the basis for randomly selecting households. Within those households a participant was selected purposively based on age. Men and women from the same household were not sampled for the focus group discussions. The results of these interviews and focus groups with patients and community members will be described elsewhere and are beyond the scope of this paper, which focuses on the perspectives of health care providers and key stakeholders within the health sector.

The quantitative data collection was undertaken in Conakry with 150 health care providers, which accounts for 11.2% of the total population of health care providers in Conakry, a sufficiently large sample size for the data from health care providers to be representative of all health care providers in Conakry. The sample included 50 doctors, 50 midwives and 50 nurses (20 of whom were auxiliary nurses), which represents 10.6% of all doctors in Conakry, 42% of all midwives, 10% of all nurses, and 4% of all auxiliary nurses in Conakry. Selection was based on a listing of all health facilities in Conakry from which the desired number of health care providers was randomly selected using a probability of selection proportional to number of each cadre of health care provider at each site. Once the desired number per site was established through this random selection process, the providers were selected using a convenience sample based on rotation schedule of providers present on the data collection days.

### Data collection and management

The data collection tools included a questionnaire for the quantitative component and interview guides for the qualitative component. The questionnaire and the in-depth interviews with health care providers, health systems managers and other health sector key informants were conducted in French.

The questionnaire included questions on knowledge, practice, and attitudes towards FGM using a questionnaire instrument developed for this study. The in-depth interviews with key informants in the health sector addressed the following themes: strategies and programmes to reduce FGM, the practice of FGM by providers, and the current training of providers on FGM. Interviews with health care providers included their perceptions of FGM (as a health care provider and as a member of the community), their training on FGM and its complications, and their perceptions of FGM being performed by health care providers.

The training of the 12 data collectors, who were medical doctors and sociologists, took place in Conakry in February 2018. The data collection tools (questionnaires, guide for FGD and in-depth individual interviews) were developed specifically for this study. Some questions for the questionnaire were adapted from existing instruments. After the data collector training workshop, a pre-test of the data collection tools was conducted in 3 sites in Conakry, which were not included in the study. Corrections were made to the data collection tools, taking into account the findings from the pre-test.

The data collection was carried out during the months of April and May 2018.

### Data analysis

The questionnaire data were entered in CSPRO 6.2, then transferred to SPSS version 20 for analysis. Descriptive data were summarized for categorical data, and bivariate analyses of relevant variables were conducted. A scale score summarizing attitudes supportive of or against FGM was created using six of the attitude measures from the questionnaire instrument and was summarized by type of health care provider.

The qualitative data from audio-recorded interviews were transcribed. Interviews conducted in Soussou, Malinké or Pulaar were translated to French. Content was analyzed twice, first using the thematic analysis approach as described by Braun and Clark [13], during which key themes were identified and categorized in line with the study objectives. The second stage of the analysis consisted of the development of a conceptual framework following Charmaz « Constructing grounded theory » [14]. In this process the identified themes from stage one of the analysis were mapped out, summarized and written up in response to the objectives of the study. Relevant quotations from the research were identified to back up major themes of the analysis.

### Ethical considerations

Written consent was obtained from the research participants prior to any audio recording of the interviews. The consent forms were translated into local languages and were submitted as part of the ethical approval process of WHO and in Guinea. Experienced data collectors were sensitized to the issue during the data collector training. The protocol of the study was approved by the national ethical committee in Guinea (099/CNERS/17 November 30, 2017) and WHO’s Ethical Review Committee (ERC 0002962v3 20/11/2017).

## Results

Table 1 shows the number of participants in the different components of the study. Forty-five in-depth interviews were conducted with health providers for this research. Table 2 shows the socio-demographic characteristics of the 150 health care providers who completed the questionnaire. The population interviewed was predominantly female (73%). Sampling covered all levels of health facilities in the city of Conakry with 39% of respondents working in national hospitals and 23% in urban health centers. Most health workers had more than 6 years of professional experience.

**Table 1:** Data collection methods and sample size.

	Questionnaire	IDI^a^	FGD^b^
**Health care providers**			
Midwives	50	8	-
Doctors (generalists and gynecologists)	50	8	-
Nurses	50	8	-
Community members			
Patients (Exit Interviews)	-	8	
Women in the community	-	-	6
Men in the community	-	-	5
Other key informants			
Health systems managers	-	6	-
Head of Health school/Medical Faculty	-	4	-
Health Professional Associations	-	3	-
TOTAL	150	45	11

^a^ In-depth interview

^b^ Focus group discussion

**Table 2:** Socio-demographic characteristics of health care providers in the quantitative component (n = 150).

		Doctors					
	Midwives	Gynecologists	Other doctors	Nurses	Nursing Assistants	Total	
	n = 50	n = 15	n = 35	n = 20	n = 30	N = 150	%
**Age (Years)**							
**< = 30**	11	1	4	1	12	29	19,3
**31–40**	17	3	11	5	9	45	30
**41–50**	15	8	12	7	5	47	31,3
**> 50**	7	3	8	7	4	29	19,4
**Sex**							
**Male**	1	12	24	0	4	41	27,3
**Female**	49	3	11	20	26	109	72,7
**Religion**							
**Muslim**	47	12	30	17	26	132	88
**Christian**	3	3	5	3	4	18	12
**Workplace**							
**National hospital**	14	11	20	9	5	59	39,3
**Communal hospital**	24	4	9	5	14	56	37,3
**Health center**	12	0	6	6	11	35	23,3
**Years of Experience**							
**< 1**	4	0	0	0	0	4	2,7
**1–2**	4	0	1	0	3	8	5,3
**2–3**	3	1	0	0	2	6	4
**3–5**	4	1	2	0	1	8	5,3
**5–10**	7	2	13	3	9	34	22,7
**10 +**	28	11	19	17	15	90	60

Table 3 shows the characteristics of the providers participating in the qualitative component. The majority were women (n = 16) and most worked in urban areas. The majority (35%) had served between 5 and 9 years. Three quarters of health care providers participating in in-depth interviews were women and a quarter were under 32 years old. Half of them worked in urban areas and a third had professional experience of 5 to 9 years. (Table 3). Key informants were mostly men and had more than 10 years of professional experience (Table 4).

**Table 3:** Socio-demographics characteristics of health care providers and other key informants in qualitative interviews (n = 20).

In-depth interviews with health care providers					
	Midwives (n = 8)	Doctors (n = 4)	Nurses (n = 8)	TOTAL	
				n = 20	%
**Age (Years)**					
Under 32	1	1	2	4	20,0
32–34	1	1	1	3	15,0
35–39	4	0	0	4	20,0
40 +	2	2	5	9	45,0
**Sex**					
Male	0	4	0	4	20,0
Female	8	0	8	16	80,0
**Location**					
Urban	4	4	4	12	60,0
Rural	4	0	4	8	40,0
**Religion**					
Christian	4	0	1	5	25,0
Muslim	4	4	7	15	75,0
Other	0	0	0	0	0,0
**Years in the hospital/health center**					
Less than 2	3	2	3	8	40,0
2–5	1	1	1	3	15,0
6–10	2	0	2	4	20,0
10 +	2	1	2	5	25,0
**Years of experience**					
Less than 5	1	0	3	4	20,0
5–9	5	2	0	7	35,0
10–14	0	0	3	3	15,0
15–19	0	0	0	0	0,0
20–24	0	0	1	1	5,0
24+	2	2	1	5	25,0
**TOTAL**	8	4	8	20	100
**In-depth interviews with other key informants**					
	Health system Managers (n = 6)	Director of Heath professional Association (n = 3)	Director of Heath Schools (n = 4)	Total	
**Age (Years)**					
Under 20	0	0	0	0	
20–24	0	0	0	0	
25–29	0	0	0	0	
30–34	6	3	4	13	
**Sex**					
Male	5	1	4	10	
Female	1	2	0	3	
**Location**					
Urban	4	3	**3**	**10**	
Rural	2	0	**1**	**3**	
**Religion**					
Christian	0	0	**2**	**2**	
Muslim	6	3	**2**	**11**	
**Years of experience**					
Under 5	1	1	0	2	
5–10	1	0	2	3	
11–15	2	0	1	3	
16–20	0	2	0	2	
20+	2	0	1	3	

**Table 4:** Social, legal and ethical aspects of attitudes about FGM based on questionnaire component.

	Midwives	Doctors		Nurses	Nursing assistants	Total	
		Gynecologists	Other doctors				
	n = 50	n = 15	n = 35	n = 20	n = 30	150	%
	%	%	%	%	%		
	**Female genital cutting/excision is a serious problem**						
Agree	98	100	88,6	95	90	141	94
Disagree	2	0	2,9	5	10	6	4
I don’t know	0	0	8,5	0	0	3	2
I do not wish to answer	0	0	0	0	0	0	0
Total	100	100	100	100	100	150	100
	**Female genital cutting/excision decreases promiscuity among women?**						
Agree	28	26,7	17,1	75	63,3	58	38,7
Disagree	66	73,3	68,6	25	26,7	81	54
I don’t know	6	0	14,3	0	10	11	7,3
I do not wish to answer	0	0	0	0	0	0	0
Total	100	100	100	100	100	150	100
	**Women who are excised are more faithful to their partners**						
Agree	22	13,4	11,4	45	60	44	29,3
Disagree	58	73,3	68,6	50	40	86	57,4
I don’t know	18	13,3	20	0	0	18	12
I do not wish to answer	2	0	0	5	0	2	1,3
Total	100	100	100	100	100	150	100
	**Female genital cutting/excision is a good practice**						
Agree	4	0	11,4	5	3,3	8	5,3
Disagree	96	93,3	85,7	95	93,3	139	92,6
I don’t know	0	6,6	2,9	0	3,3	3	2
I do not wish to answer	0	0	0	0	0	0	0
Total	100	100	100	100	100	150	100
	**Female genital cutting/excision is a violation of women’s and girls’ rights**						
Agree	96	86,7	80	100	83,3	134	89,3
Disagree	2	13,3	5,7	0	13,3	9	6
I don’t know	2	0	11,4	0	3,3	6	4
I do not wish to answer	0	0	2,9	0	0	1	0,7
Total	100	100	100	100	100	150	100
	**Female genital cutting/excision should be criminalized**						
Agree	96	80	57,1	85	76,6	120	80
Disagree	4	13,3	22,8	10	16,6	19	12,6
I don’t know	0	6,6	14,2	5	6,6	9	6
I do not wish to answer	0	0	5,7	0	0	2	1,3
Total	100	100	100	100	100	150	100
	**Attitude scale (0–6) measuring attitudes towards FGM. Higher response corresponds to less supportive of FGM**						
< = 3	2	13	14	0	7	10	7
4	10	7	14	20	23	22	15
5	30	33	20	65	50	55	37
6	58	47	51	15	20	63	42
	**It is possible to end female genital cutting/excision within one generation**						
Agree	96	86,6	80	100	90	136	90,6
Disagree	0	6,6	17,1	0	10	10	6,6
I don’t know	4	6,6	2,8	0	0	4	2,6
I do not wish to answer	0	0	0	0	0	0	0
Total	100	100	100	100	100	150	100
	**Health care providers who perform female genital cutting/excision are violating medical ethics**						
Agree	92	86,7	85,7	95	90	135	90
Disagree	4	0	5,7	5	6,7	7	4,7
I don’t know	4	13,3	2,9	0	3,3	6	4
I do not wish to answer	0	0	5,7	0	0	2	1,3
Total	100	100	100	100	100	150	100

### Attitudes of health providers on FGM in general

**Fig 1:**
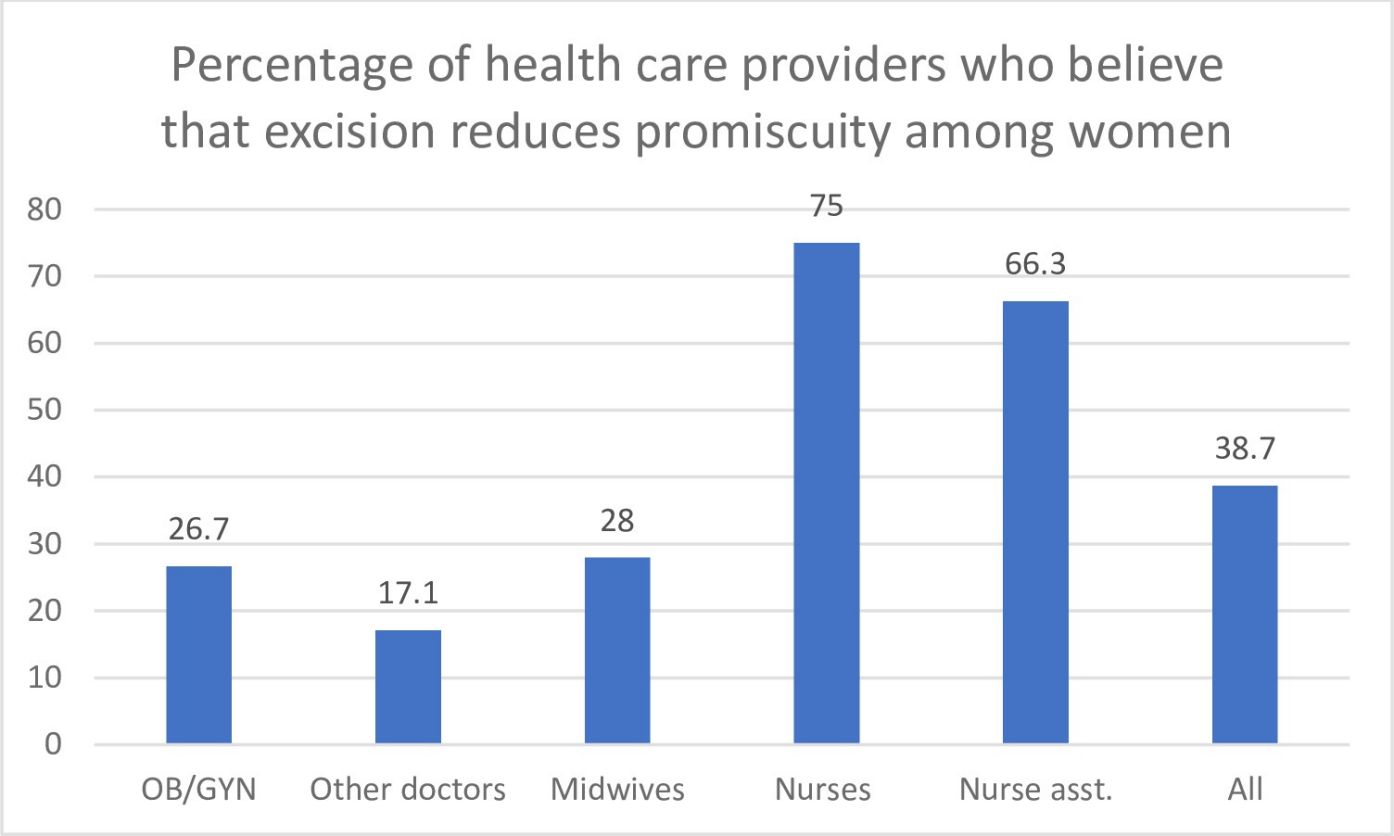
Percentage of health care providers who believe that excision reduces promiscuity among women.

Findings from the questionnaire revealed that 94% of health care providers in Conakry think that FGM is a serious problem. Only 4% say that it was not (Table 4). However, more than one-third of health providers (38%) think that FGM decreases the risk of promiscuity among women. This belief is much more pronounced among nurses (75%) and auxiliary health providers (63%) (Table 4, Fig 1) as compared to doctors (17%). Results of an attitude scale show that the majority of the 150 health care providers interviewed reported attitudes not supportive of FGM, with almost half (46%) reporting non-supportive attitudes on all six attitude questions and 37% reporting non-supportive attitudes on five of the six domains. The attitude scores tended to be consistent across health care provider cadres, but some variation by type of health care provider can be noted. Specifically, more midwives had six responses that indicated they were not supportive of FGM, while more doctors had three or less responses not supportive of FGM. However, the sample size by type of provider was not adequate to assess the statistical significance of differences between the groups.

The analysis of the qualitative component showed that the majority of midwives interviewed reject FGM (Labé and Faranah) while a minority support it. The midwives who support the practice suggest that it is performed to avoid stigmatization, to temper sexual desire and to prevent promiscuity of girls. The following quote is from a midwife from a rural area of Labé:

*"There are some who say that if we do not excise, the girls become vagabonds in life"*.

Health systems managers also reported this reason:

*“You know that in our cultures [in Guinea], according to our social etiquette, the practice has always existed. That is why people who reason in that way would continue with the practice. It is a custom and it is a custom–that is why we need to do it! The second reason […] is that friends or colleagues would make fun of someone. At some point in a workshop someone explained to them [to stop practising FGM], and afterwards they all went to wash and when one of them [a woman] took her clothes off saying that she had not been excised, although she had been, everyone made fun of her. Maybe another reason is that it [not excising a girl] leads to her not really getting[sexual] satisfaction, because she feels like having sex all the time if she’s not excised. So in order to reduce that, people think that girls need to be excised, otherwise she’d fall in love and there are many other problems linked to that.”*

**Fig 2:**
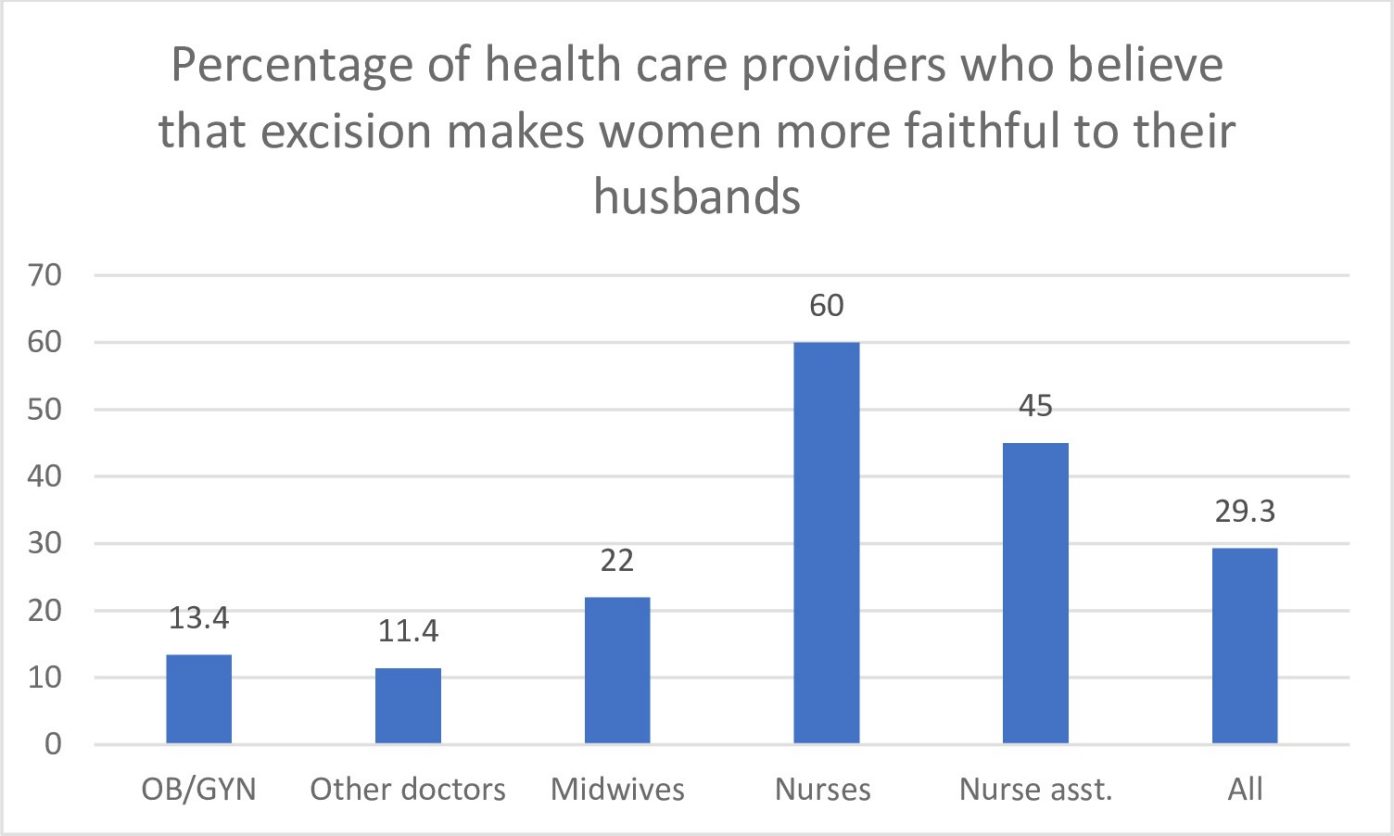
Percentage of health care providers who believe that excision makes women more faithful to their husbands.

As seen in this quote, customs and traditions are highly cherished and guide people in their moral conduct, regardless of whether the tradition is also a harmful practice. Thus, practices that are perceived as tradition are endorsed as being closely linked to social etiquette, morality and ethnic identity. It is believed that a woman would be mocked, ridiculed and socially excluded if it became public knowledge that she is not cut. Furthermore, it is believed that an uncut woman cannot control her sexual appetite, which is why it needs to be tempered. In the quantitative component, the majority of respondents (57,4%) disagreed with the statement that excised women are more faithful than those not excised, but a third of health care providers (29%) agreed with it. This belief is also much more pronounced among nurses (45%) and auxiliary nurses (65%) (Table 4, Fig 2).

Moreover, this study shows that some health care providers still maintain that FGM is a good practice (11% of doctors, 4% of midwives, 5% of nurses and 3% of auxiliary nurses (ATS) in Conakry). While 86% of doctors, 96% of midwives, 95% of nurses and 93% of auxiliary nurses are against the practice of FGM, (Table 4), the interviews revealed that some midwives in rural Faranah think it is important to excise girls to prevent them from being stigmatized, showing some ambivalence about the practice.

All of the midwives participating in in-depth interviews state that if asked for their opinion on girls’ excision, they will not recommend it because of the health conditions that they have observed in excised women, such as bleeding, pain, loss of consciousness and loss of sexual sensitivity. In the opinion of some nurses, excision was a good practice in the past, but that now it was considered a bad practice and even a crime. They claim that a family insisting on having their daughters excised could face criminal penalties.

*"Excision, what I hear about excision is that excision means cutting, cutting the clitoris. FGM is a crime for me."*

This was also found in the quantitative component, in which 80% of healthcare providers believed that the practice of FGM should be criminalized. These findings varied according to the professional profiles of the health care providers: 57% of doctors surveyed and 96% of midwives surveyed agreed with this statement (Table 4).

Health providers state that they seek training on the management of the complications of FGM and on how to reject demands for excision.

### Providers’ attitudes towards the abandonment of FGM

A large number of health providers surveyed in Conakry believe that it is possible to end FGM within a generation, while a minority think the contrary. The latter point of view is much more pronounced among doctors (17%.) (Table 4). In addition, the results of individual interviews with health care providers suggest that in order to achieve the abandonment of FGM, it is necessary to raise awareness about the practice at the societal level because in their view, there is a lack of understanding about what FGM is and what it entails. There is also an understanding that the law can only be effective with education. One healthcare provider explained:

*"It is consensus that we favor. We should always raise awareness, explain, because here, the problem is a problem of understanding that arises at the level of society. The people who come to explain, are people who believe that they know what they are talking about and as soon as we do that, some illiterate people block up. Even if they wanted to do it [stop practicing] they may refuse because you were maybe (arrogant) towards them. With all this, referring to justice should be the last resort. It is necessary to sensitize them 1000 times to explain it 1000 times before taking a step towards the judicial system."*

### Attitude of health care providers to the medicalization of FGM

It is important to note that health care providers recognize that excision is also practiced by health workers in Guinea. Indeed, the respondents stated that the profile of persons practicing excision in Guinea was traditional excisers (88%), followed by midwives (53%) and nurses (27%) (Table 5). The medicalization of FGM is also reported by health providers in individual interviews without detailed specification on the type of profile of the health-worker who performs it.

**Table 5:** Profile of the person who performs excision (%).

	Midwives	Doctors			Nurses	Nursing assistants	Total (n = 150)	
			Gynecologist	Doctors				
	n = 50		n = 15	n = 35	n = 20	n = 30	Number	%
	%		%	%	%	%		
	Who performs excision in your country (select all that apply)							
**Traditional excisor**	96		100	94,2	70	73,3	132	**88**
**Medical doctors**	0		0	11,4	0	6,7	6	**4**
**Nurses**	32		13,3	25,7	10	40	41	**27,3**
**Midwives**	54		66,6	31,4	60	66,6	80	**53,3**
**Grandmother or other family member**	4		6,7	2,8	5	6,6	7	**4,6**
**Matron**	2		0	0	10	0	3	**2**
**Don’t know**	0		0	0	1,3	0,7	3	**2**

Almost all health care providers (90%) believed that the practice of excision performed by health care providers violates medical ethics with little variation in the percentage according to the profiles of health workers ranging from 95% of nurses to 85% of doctors (Table 5). In the qualitative interviews, medical ethics were not explicitly mentioned by the health care providers. Their opposition to excision was essentially based on the fact that it is prohibited by law and that excision can lead to complications. The following quote from a midwife from an urban area illustrates this point:

"*Because health workers are afraid*, *they hear talk of it on the radio*, *people talk about it*. *If you get arrested for that you are screwed*.

For some health system managers, the medicalisation of FGM is preferable to the practice being performed by traditional excisers due to harm reduction attempts by performing the practice under hygienic conditions.

"*Well*, *it’s not a good practice*, *but it’s important to recognize that there’s less risk*, *when it’s done by health professionals*. *When it is done by the traditional excisers here*, *there are sometimes terrible consequences*, *bleeding*, *there is also the risk of transmission of diseases*. *I said that sometimes we all have to roll up our sleeves and decide to sanction those who are not in agreement with banning the practice*. *But I believe that for this practice to end*, *everyone needs to get involved*, *because it is a social phenomenon*. *At every level*, *everyone has a role to play if we want to achieve*.

### Attitude of health providers faced with a request for excision

The majority of providers stated that health providers usually refuse requests from families seeking excision. Some health providers in rural areas believe that a health worker would agree to excision because it is a custom or because of lack of information. A midwife in a rural area said:

*"There are some who accept and others who refuse. But the majority refuse now, because there is information available on that, provided by WHO"*

According to most health system managers, health care providers agree to perform FGM because of social relations and because of the money and the status and power it confers.

*“in the countryside, when I lived there, it was a financial problem, a problem of need and a problem of notoriety [being a local celebrity] because the one traditional exciser who did it there was of a certain social hierarchy. Secondly, the families felt that if she did the excision of their daughter, they would bring her a lot of things in return. […] So the exciser had some power, and she received good pay, which strongly encouraged the excisers to maintain the practice.”*

Although no formal payment was necessarily requested for performing FGM, according to local customs of solidarity and exchange, individuals would feel obliged to show their gratitude in kind, i.e., by giving whatever is available from the farm, such as crops, millet, peanuts, rice, dried beans, mais, etc.

When asked what they felt drives families to ask providers to excise their daughters, some health systems managers cited customs, social pressure and ignorance of the negative consequences of the practice.

*“What pushes them is that it is a tradition, a bad custom in society, such as ignorance …how can I say it is a form of imitation that pushes people to practice mutilation. If all the other girls undergo it, why not my girl? Because if it is not done, I will be ashamed, I will be looked at differently everywhere I go, therefore I conform to the society.”*

Besides financial interest, solidarity towards kin also emerged as a driver for medicalized FGM as seen in the quote from another health system manager:

*“well it is true that there is the lucrative aspect that people receive something when they perform it, that’s a reality. But it is also certain, I would say weakness too, because when it is a parent who asks you to carry it out, sometimes you don’t perform it for money, for example, because this sister, that niece, asked you to perform it on her girl, sometimes we do that too, in order to serve the parent, family or friends, sometimes there are some who do it because of that so these are two fundamental reasons. There is the financial side and there is also the personal side, to serve people.”*

## Discussion

FGM prevalence remains very high in Guinea. Demographic Health Survey data from 1999 until 2018 show that the prevalence of the practice has only decreased from 99% to 95% in 20 years, although further declines are noted in younger age cohorts with 92% of girls aged 15–19 having been excised according to the most recent estimates of DHS, 2018. The previous surveys also show that whereas in DHS 1999, only 9% of adult women were excised by a health professional, in DHS 2018 this figure has risen to 18% among 15–19 year old women, 37% report having been cut by health care providers according to data from 2018. It is striking that after more than 20 years of sustained effort to bring the practice to an end the medicalization of FGM is clearly on the rise in Guinea [10, 15].

This mixed methods study showed several important findings. Specifically, this study provided an in-depth glimpse into the attitudes towards FGM within the health system—not only health care providers but also decision-makers within the health system as well as patients and community members who interact with the health system. The findings have important implications for practice and for how to strengthen the health system by revealing the need to address not only clinical management skills but also to ensure that the providers themselves do not perpetuate attitudes and practices supportive of FGM.

### Accountability and the law

Our study showed that nine out of ten health care providers think that FGM performed by health-providers violates medical ethics. Furthermore, three-quarters of the health care providers interviewed describe FGM as a crime. Similar findings have been found in other high prevalence settings [14]. This study also revealed that health professionals in Guinea think that the laws are insufficient and should be enacted in conjunction with sensitization campaigns, and community and patient education programs. Similar views have been expressed by health professionals in other studies based in Sub-Saharan Africa [16] and legal scholars have stated that criminal law without community awareness raising is not a sufficient mechanism for change [17].

Our results also show that for some health care providers, the law was a deterrent. However, additional accountability mechanisms within health professional associations, such as losing one’s professional license, incurring fines, and other penalties if providers perform FGM were currently lacking.

Some health-professionals in this study (11,3%) did not know the law criminalizing the practice or were not certain about the content of the law and their duties with regard to these laws. In contrast, a study in the Gambia, a country with low rates of medicalization, showed that the majority of health professionals (75,4%) support the law criminalizing FGM [18]. The same study also found that three quarters of health care providers considered it a dangerous practice and wished for its abandonment [18]. The negative impact of this practice on women’s health and well-being as well as it being a human right violation are cited as the main reasons in support of abandonment [18].

### Attitudes driving medicalization

FGM is performed despite the fact that families and health professionals are aware of the known consequences of the practice; nevertheless the social advantages of FGM are perceived as being more important than the risks and complications [19]. In an integrative review of studies exploring the motivations of health care providers in performing FGM, Doucet et al identified four main reasons why health care providers carry out FGM. These included the fact that providers were from the same practicing communities and were subject to the same social norms, that they were satisfying a community demand for the practice, the belief that medicalized FGM would reduce harm, and that there was a financial incentive in performing the practice [20]. Likewise, the current study found similar beliefs driving the practice of medicalized FGM as reported by study participants.

The qualitative component of the study shows that the majority of interviewed midwives reject excision due to risk of complications. However, some suggested that medicalized FGM was safer than when performed by a traditional exciser. Such perceptions of medicalized female genital cutting for harm reduction strategies have been extensively debated over the last 20 years [21–23]. WHO and other UN agencies continue to oppose medicalized FGM because it violates medical ethics, is contrary to multiple human rights principles and implies a tolerance for a practice that is harmful.

In this study, some health care providers suggested that FGM reduces the risk of debauchery in girls and women by diminishing their sexual desire. The preservation of virginity before marriage is highly valued across Guinea, and FGM is perceived as a way to help girls temper their sexual desires before marriage. The research showed that the sexual enjoyment and promiscuity of women is equated with prostitution and the demise of civilization, rather than being perceived as a right. Sexual norms of males were not mentioned showing the pressure placed on girls and women to maintain social order in terms of controlling sexual behavior, which is not equally expected from their male counterparts. Other authors found similar reasons for the tolerance towards FGM among health professionals [15, 24]. As in the current study social pressure is another important factor mentioned as leading to the continuation of the practice [24].

The material and financial gains of FGM emerged as an important motivation of health workers in performing the practice. Financial gain as a motivating factor has been described extensively in other studies too [20, 25, 26]. However, it needs to be said that the cost of medicalized excision in Guinea is not high and the sum is symbolic rather than fixed. Some of the interviewees described those who perform the practice as receiving in-kind gifts. Although receiving gifts of food is not negligible in terms of subsistence for underpaid health staff, there is not clear evidence that this symbolic pay is driving increased medicalization.

### Need for FGM content in the training of health providers

According to existing literature, lack of information on FGM, and lack of training of providers, is an important aspect of the rise in medicalization [20, 24]. A systematic review on the attitudes and experiences of health providers identified lack of knowledge and lack of training on FGM as common challenges that providers are faced with [24]. Similar to the review’s findings, the current study found that knowledge gaps on FGM, such as not detecting FGM, not knowing the complications associated with the practice, and not offering the appropriate care and support to women who have undergone FGM were also common in Guinea. Health care providers in our study noted the absence of directives or detailed guidelines on how to report cases and complications linked to FGM. In a systematic review, Zurynski et al, note that health professionals need practical guidance as well as training on how to prevent FGM and manage its complications [27].

### Strengths and limitations

This is the first study exploring the beliefs, attitudes and practices regarding FGM and its medicalization in Guinea using a mixed methods methodology with participants in a range of roles within the health system. Previous studies on FGM in Guinea presented secondary analysis of population-based studies [28]. This study makes an important contribution in showing the challenges that the health system is facing in responding to FGM in Guinea. Given the sensitive nature of the topic and illegality of the practice, none of the health professionals interviewed admitted to ever having performed FGM. In fact they were not directly asked about their own involvement in the practice, which can be considered a limitation of this study.

Likewise, this study covers only three of Guinea’s eight administrative regions. Given the country’s rich ethnic diversity and cultural variations there may be other factors influencing the medicalization of FGM that were missed in this research, therefore the results cannot be generalized beyond these study regions. Despite these limitations, some of the results are relevant nationally. For example, there are no national guidelines on the clinical management of FGM and no content in the training curricula of nurses, midwives and doctors on FGM and how to manage complications and provide support to women and girls who are affected by this harmful practice.

### Implications for research and practice

The research presented here has been informative in describing some important perceptions of health care providers, decision-makers and patients within the health system as they relate to FGM. These findings are relevant in developing strategies to strengthen the role of the health system in addressing FGM. In particular, this research explored the attitudes of health care providers regarding FGM and its medicalization; the need for accountability mechanisms to prevent the medicalization of FGM as it constitutes a violation of medical ethics; the role of the law in prohibiting the practice including by health care providers; and understanding the weaknesses in the health system in training health care providers on how to provide treatment and care to women and girls who have undergone the practice.

The findings of this research have been applied in several concrete ways. First of all, they were presentated at the outset of a national workshop to develop a national action plan for health sector on FGM and have informed the development of the national action plan. In addition, this research has also informed the development of an intervention package to improve communication between providers and the community around FGM prevention in the context of antenatal care service provision for Guinea and other high prevalence countries. This package is currently being tested in a Phase 2 of this study [12].

The study also showed the need for greater integration of FGM content into pre-service and in-service trainings of health care providers, and the need for national guidelines and policy directives from the health sector. These activities have already been initiated in Guinea following this research, which has led to the development and strengthening of the health sector approach to FGM as part of a multi-sectoral approach to abandonment of this harmful practice in the country.

Improving the knowledge and skills of health care providers on FGM in general is an important step in reducing medicalization. The health system can play an important role in promoting the abandonment of FGM and particularly of its medicalization. Having a supportive policy framework in addition to the law is an important step in ensuring that health care providers have the guidance and support they need when faced with community members who request the practice. These activities must go hand in hand with other activities working with affected communities.

## Conclusion

The study reveals the important role of health care providers in the prevention and abandonment of FGM. The absence of national guidelines and of adequate training on FGM prevention and care as well as lack of knowledge about the law, and strong social pressure in support of FGM contribute to the beliefs and attitudes held by health care providers. By understanding the attitudes of health care providers, training programs and other initiatives will be better able to address these underlying beliefs and norms and improve the way that the health system provides care and prevention services for FGM.

## Supporting information

S1 Data (XLSX)

S2 Data (XLSX)

S1 File Questionnaire for health care providers. (DOCX)
