# Supplementary material for: Attitudes of health care providers regarding female genital mutilation and its medicalization in Guinea
Source: PLoS One. 2021 May 13;16(5):e0249998. doi: 10.1371/journal.pone.0249998 (PMC8118326; doi:10.1371/journal.pone.0249998)
Supplement: S1 File — (DOCX) [file pone.0249998.s003.docx]

S1 File. Questionnaire for health care providers

**SURVEY QUESTIONNAIRE FEMALE GENITAL CUTTING/EXCISION**

Date: ___ / ___ / ___

Survey objective: To explore female genital cutting/excision -related knowledge, attitudes, and practices among health-care providers in _____________________________________.

*Information to read to respondent:*

We wish to learn about your knowledge, attitudes and practices regarding female genital mutilation/cutting. We hope to understand the best way to bring information to you and to……………………………………... The information you provide will be used to improve the training of healthcare providers. Your answers will not be released to anyone and will remain anonymous. Your name will not be written on the questionnaire or be kept in any other records. Your participation is voluntary and you may choose to stop the interview at any time.

Thank you for your assistance.

**Interviewer:** Place an X in the box of the selected answer(s).

Do not read responses unless the directions indicate

GENERAL AND DEMOGRAPHIC QUESTIONS

1. **What is your age?**

1. ☐ Under 30

2. ☐ 31–40

3. ☐ 41–50

4. ☐ Over 50

1. **What is your sex?**
2. ☐ Female
3. ☐ Male
4. **What is your country of origin?**

_________________________________________________

1. **What is your religion?**

☐ Muslim

☐ Christian

☐ Other

1. **What is your occupation/designation?**

☐ Midwife

☐ Nurse

☐ Physician. *Specify your specialty* ______________________

☐ Other. *Specify* _____________________________________

1. **Where do you work?**

☐ University Hospital

☐ Rural hospital

☐ Community health centre

☐ Private hospital/clinic

☐ Family planning clinic

☐ Other. *Specify* __________________________________________

1. **For how many years have you been working in your field?**

☐ Less than 1 year

☐ Between 1 year and 2 years

☐ Between 2 years and 5 years

☐ Between 5 years and 10 years

☐ Over 10 years

A. KNOWLEDGE

1. **Have you ever heard about female genital cutting/excision?**

☐ Yes

☐ No

1. **Do the women in your community undergo female genital cutting/excision?**

☐ Yes

☐ No

☐ I don’t know

1. **How many categories/types of female genital cutting/excision are you aware of?**

☐ None

☐ One

☐ Two

☐ Three

☐ Four

☐ Other. *Specify* _____________________________________

1. **Can you list three possible health complications from female genital cutting/excision?**

1. _____________________________________________________

2. _____________________________________________________

3. _____________________________________________________

☐ No

☐ Refused/no answer

1. **Is female genital cutting/excision illegal in your country?**

☐ Yes

☐ No

☐ I don’t know

1. **Who carries out female genital cutting/excision in your country? Tick the two main people who do this in your country.**

☐ Traditional circumciser

☐ Doctors

☐ Nurses

☐ Midwives

☐ Grandmothers or other female family members

☐ Other. *Specify:* ________________________________________________

☐ I don’t know

1. **Are you aware of official guidelines on the management of female genital cutting/excision and its complications?**

☐ Yes. *If yes, please specify which ones ____________________________________*

☐No. *Go to question 16.*

1. **Have you read them?**

☐ Yes

☐ No

☐ I am not aware of them

1. **If after discussing with or examining a woman you confirm she has an infibulation (type III female genital cutting/excision), should you offer her/ refer her for a deinfibulation (surgical opening of infibulation)?**

☐ Yes

☐ No

☐ I don’t know

1. **Are women with female genital cutting/excision at increased risk of negative obstetric outcomes (e.g. C-section, perineal tears, increased blood loss during delivery)?**

☐ Yes. *Specify why___________________________________________________*

☐ No. *Specify why not___________________________________________________*

☐ I don’t know

1. **If a woman with infibulation (type III female genital cutting/excision) has had de-infibulation (surgical opening of infibulation) to give birth, should you re-stitch/re-infibulate her once the baby is born?**

☐ Yes, always. *Specify why____________________________*

☐ Yes, if the patient or her family/partner request this

☐ Never. *Specify why____________________________*

☐ I don’t know

1. **Can genital cutting decrease sexual pleasure in women?**

☐ Always

☐ Sometimes

☐ Never

☐ I don’t know

1. **Can genital cutting affect the mental health and well-being of women and girls?**

☐ Always

☐ Sometimes

☐ Never

☐ I don’t know

1. **When you treat or attend a girl or a woman with genital cutting, do you think you have enough knowledge/skills to provide good quality health care?**

☐ Yes

☐ No

☐I don’t know

1. **During you pre or post graduate training, did you receive any formal training on female genital cutting/excision?**

☐ Yes.

☐ No. *Go to section B*

☐ I don’t know

1. **When did you receive the training?**

☐ During my studies (pre-service training)

☐ After graduation/at work (in-service training)

☐ I don’t know/remember

☐ Not applicable

1. **What was the format of the training? (Check all that apply)**

☐ Classroom lessons/seminars

☐ Workshops

☐ Digital format (E-learning videos; smart phone app)

☐ During clinical practice under supervision of a mentor

☐ Other. *Specify* _____________________________________

☐ Not applicable

B. ATTITUDES

**For each of the following statements please state if you agree/disagree/ don’t know or do not want to answer.**

1. **Female genital cutting/excision is a serious problem**

☐ Agree

☐ Disagree

☐ I don’t know

☐ I do not wish to answer

1. **Health care providers who perform female genital cutting/excision are violating medical ethics**

☐ Agree

☐ Disagree

☐ I don’t know

☐ I do not wish to answer

1. **Health care providers who perform female genital cutting/excision should be sanctioned**

☐ Agree

☐ Disagree

☐ I don’t know

☐ I do not wish to answer

1. **Female genital cutting/excision decreases promiscuity among women?**

☐ Agree

☐ Disagree

☐ I don’t know

☐ I do not wish to answer

1. **Women who are excised are more faithful to their partners**

☐ Agree

☐ Disagree

☐ I don’t know

☐ I do not wish to answer

1. **Female genital cutting/excision is a good practice**

☐ Agree

☐ Disagree

☐ I don’t know

☐ I do not wish to answer

1. **Female genital cutting/excision is a violation of women’s and girls’ rights**

☐ Agree

☐ Disagree

☐ I don’t know

☐ I do not wish to answer

1. **Female genital cutting/excision is a religious mandate**

☐ Agree

☐ Disagree

☐ I don’t know

☐ I do not wish to answer

1. **Female genital cutting/excision should be criminalized**

☐ Agree

☐ Disagree

☐ I don’t know

☐ I do not wish to answer

1. **It is possible to end female genital cutting/excision within one generation**

☐ Agree

☐ Disagree

☐ I don’t know

☐ I do not wish to answer

1. **Would you like to receive more training on female genital cutting/excision?**

☐ Yes

☐ No

☐ I don’t know

☐ I do not wish to answer

C. PRACTICE

.

1. **If a pregnant woman with genital cutting is expected to have a girl, do you discourage her from having her daughter cut?**

☐ Yes

☐ No

☐ I don’t know

1. **If your patient is pregnant and has infibulation (type III female genital cutting/excision), do you offer deinfibulation?**

☐ Yes

☐No

☐I don’t know

1. **Do you counsel/brief your female patient about the deinfibulation, specifically about the surgery and the changes this will cause?**

☐ Yes, before the deinfibulation

☐ Yes, after the deinfibulation

☐ Yes, before and after the deinfibulation

☐ No

1. **Do you counsel/brief the women’s partner about the deinfibulation, specifically about the surgery and the changes this will cause?**

☐ Yes, before the deinfibulation

☐ Yes, after the deinfibulation

☐ Yes, before and after the deinfibulation

☐ No

1. **If a woman with infibulation (type III female genital cutting/excision) has had de-infibulation (surgical opening of infibulation) to give birth, do you perform re-stitching/re-infibulation once the baby is born?**

☐ Yes, always. *Specify why____________________________*

☐ Yes, if the patient request this

☐ Yes, if the patient’s family or partner request this

☐ Never. *Specify why____________________________*

1. **If you hear of or see a colleague performing female genital cutting/excision, what do you do?**

☐ I report him/her to the authorities

☐ I discuss with him/her and explain to him/her that health care providers should not perform female genital cutting/excision

☐ I do not get involved

☐ I don’t know

1. **How often do you look for female genital cutting/excision when performing a gynaecological examination of the vulva?**

☐ Always

☐ Often

☐ Sometimes

☐ Rarely

☐ Never

1. **How often do you record the female genital cutting/excision in the women’s medical file?**

☐ Always

☐ Often

☐ Sometimes

☐ Rarely

☐ Never

1. **If a girl comes with acute health complications after being cut/excised, do you report the issue to the authorities?**

☐ Always

☐ Often

☐ Sometimes

☐ Rarely

☐ Never

1. **What measures would be effective to discourage health care providers from performing female genital cutting/excision?**

________________________________________________________________________________________________________________________________________________________________________________________________________________________________________________________________________________________________________________________________________________________________________

**2. In-depth interview guide health care providers (physicians, midwives and nurses)**

**A Health systems response to FGM and it medicalization**

**Formative research**

**PHASE 1: FORMATIVE RESEARCH**

***__________________________________________________________________________________***

**In-depth interview guide**

**Health care providers (Physicians, midwives and nurses)**

**Step 1:** Introduce yourself to the participant. Describe the purpose of the interview and how information will be used. Obtain informed consent.

**Step 2:**  Ask the participant to complete the table below on socio-demographic information prior to beginning the interview.

**Step 3:**  Conduct the interview. Please remember to audio record the interview if participant agrees.

***Participant information:***

**Age:**

**Sex:**

**Highest level of education:**

**Profession:**

**Name of health facility employed at:**

**Cadre and position:**

**Number of years of experience in that facility:**

**Number of years of experience in total:**

**Ethnic group:**

**Religion:**

**Interview date:**

**Start time:**

**End time:**

**Interviewers:**

**A. PERCEPTIONS OF HEALTH CARE PROVIDERS REGARDING FGM (as member of the community and as health-care provider)**

1. What do you know about excision (FGM)? Have you heard people talk about it?
2. What do you think about excision? What does it represent to you?
3. As a health care provider, do you feel that it is important to excise girls? Why or why not?
4. Do you know families in your community who do not practice excision? (Ensure that the participant understands that you are not asking for names of families.)
   1. If yes, how are they viewed by others in the community?

B. CASE STUDY 1 (Midwives and nurses)

Now I am going to read you a short description of a situation that you might face. Please remember that the names have been invented and this is not a true story.

Agnes is a 45 year old housewife who was excised when she was a child. Her older son Paul married Ella 3 years ago and they now have a 2 year old daughter, Ida. Agnes has come to discuss with you because she is concerned her son and daughter in law do not want to excise Ida. They say they fear she might have medical complications and do not want to hurt their daughter. Agnes would like to know your opinion as a trained midwife and asks you if there would be any way of minimizing the risks during the excision.

1. What do you tell Agnes?

2. What do you do?

CASE STUDY 2 (Doctors)

Now I am going to read you a short description of a situation that you might face. Please remember that the names have been invented and this is not a true story.

Yemeni is a 20 years old housewife. At the age of 6 she was infibulated. She has been married for 2 years and is very happy because she is now pregnant with her first baby. Becoming pregnant was not easy and very painful for Yemeni. She and her husband felt intense pain during sexual intercourse due to her infibulation. Yemeni knows that all women who are infibulated and give birth must be opened in order to allow the passage of the baby, but she is very scared of this situation. During her first antenatal care visit she has requested to speak to a doctor so she can ask more details about the deinfibulation procedure, and specifically about what will happen to her once she has given birth.

1. How do you explain the deinfibulation procedure to Yemeni?

2. What to you tell Yemeni will happen after she has given birth?

**C. TRAINING ON FGM AND HEALTH COMPLICATIONS**

1. During your professional education, did you receive information about FGM (i.e., types of FGM) and any training on treating health consequences from FGM)? Explain.
   1. If yes, have you applied the information you gained from this training?

1. Do you generally discuss FGM with your clients who have undergone FGM?
   1. If yes, how do you feel when you talk to your clients about FGM? Have you experienced difficulties in discussing FGM with clients?
   2. If not, why not?
2. What training would be essential for you to manage complications related to FGM?
3. What would be your preferred format for receiving this training?
4. In general, when you need to update your knowledge on FGM, what sources do you consult and which do you consider the most reliable?

**D. FGM PERFORMED BY HEALTH CARE PROVIDERS**

1. Sometimes a women’s family members may request that health care providers perform FGM. What is your view on FGM being carried out by health care providers? Please explain.
2. In your opinion why do families send their daughters to health care providers to undergo FGM?

To your knowledge, do healthcare provider usually accept these requests or do they oppose these requests?

Why do you think they accept or oppose these requests?

1. What can a healthcare provider do if he/she is asked to perform FGM and he/she does not wish to carry out the procedure?
   1. What can he/she tell the family?
   2. Who can she/he reach out to?
   3. What other actions can he/she take?

**4. In-depth interview guide professional associations**

**Formative research**

**PHASE 1: FORMATIVE RESEARCH ___________________________________________________________________________**

**In-depth interview guide**

**Professional associations**

**Step 1:** Introduce yourself to the participant. Describe the purpose of the interview and how information will be used. Obtain informed consent.

**Step 2:**  Ask the participant to complete the table below on socio-demographic information prior to beginning the interview.

**Step 3:**  Conduct the interview. Please remember to audio record the interview if the participant agrees.

**Socio-demographic characteristics of participant**

| **Participant’s**  **Profession #** | **Age group** (circle) | **Sex** (circle) | **Cadre and position**  **in the facility**  (write in) | **Total years of service**  **in years**  (circle) | **Years of service in current facility** (circle) | **Religion** | **Ethnicity** |
| --- | --- | --- | --- | --- | --- | --- | --- |
|  | <25  25-34  35-44  45 + | Female  Male |  | < 1  1 – 5  6-14  Over 14 y | < 1  1 – 5  6-14  Over 14 | Muslim  Christian  Other |  |

**SITE: -------------------------------------- Urban/Rural**

**Facility: Health center**|___|  **Hospital**|___|  **Private clinic/Center**|___|

**Start time:**

**Interviewer ID __ __ __ __**

**Interview date: __ __/__ __/__ __**

DD / MM / YY

**A. INTRODUCTORY QUESTIONS**

1. What is your role in the institution?
2. What are the main functions of this professional association?
3. We understand that health care providers are increasingly practicing FGM. What do you know about this phenomenon?

**B. FGM PERFORMED BY HEALTH CARE PROVIDERS**

1. In your opinion, what are the reasons that despite the awareness raising campaigns and bans, some health care providers continue to practice FGM? What do you think are their motivations?

**C. PROFESSIONAL RESPONSIBILITY AND CODES OF CONDUCT AGAINST FGM**

Now I would like to discuss codes of conduct and how health care providers adhere to them in Guinea.

1. Are you aware of existing strategies that have been put in place in Guinea to ensure that health care providers adhere to existing codes of conduct as they relate to FGM?
   1. If yes, have any of these strategies been put in place by your professional association?
2. In your opinion are these strategies to enforce the adherence to the existing codes of conduct effective in ensuring that providers do not carry out FGM? Explain?
3. What are the punitive consequences for health care providers who carry out FGM?
4. Do you think that health care providers fear these consequences and respect the measures put in place to prevent FGM being carried out by providers? Explain?
5. How could these strategies be strengthened in order to improve the adherence of health care providers to existing codes of conduct?
6. In Guinea, numerous laws have been put in place to fight against FGM, however FGM continues. How do you feel that the actions of your association could have a meaningful effect on the prevention of FGM?
7. Do you feel that a teaching curriculum for health care providers that addresses medicalization of FGM would be useful or relevant in Guinea?

**D. TEACHING PROGRAM ON FGM**

Now, I would like to ask you a few questions about the teaching programs on FGM aimed at health care providers that have been implemented in Guinea.

1. Are you aware of teaching programs on FGM aimed at medical, nursing or midwifery students? (*Interviewer should indicate the type of provider*)

If yes,

- 1. Have these teaching programs been evaluated? Based on evaluations and your perceptions,
     - 1. Are these programs correctly administered (i.e., administered in a standardized way)?
       2. Is the content appropriate?
       3. Is the content and format acceptable to providers?

If no,

1. How feasible would these programs be to implement?
2. What potential challenges would you expect with the implementation of these programs?
3. Finally, what other strategies/programs would you suggest putting in place to reduce FGM by medical personnel (e.g., training, communication strategies (describe content and channels), follow up, evaluation)?

**5. In-depth interview guide Health systems managers**

**A Health systems response to FGM and it medicalization**

**Formative research**

**PHASE 1: FORMATIVE RESEARCH __________________________________________________________________________________**

**(Prefectural health directors, Regional health directors, Hospital Directors, Health center Head)**

**Step 1:** Introduce yourself to the participant. Describe the purpose of the interview and how information will be used. Obtain written consent.

**Step 2:**  Ask the participant to complete the information below on socio-demographic information prior to beginning the interview.

**Step 3:**  Conduct the interview. Please remember to audio record the interview.

**Socio-demographic characteristics of participant**

**Age:**

**Sex:**

**Highest level of education:**

**Profession:**

**Name of health facility employed at:**

**Cadre and position (write in):**

**Number of years of experience in that facility:**

**Number of years of experience in total:**

**Ethnic group:**

**Religion:**

**Interview date:**

**Start time: End time:**

**Interviewers:**

**SITE: -------------------------------------- Urban/Rural**

**Facility: Health center**|___|  **Hospital**|___|  **Private clinic/Center**|___|

**A. INTRODUCTORY QUESTIONS**

1. What is your role in this institution?
2. Since when have you had this position?
3. What are the main functions you carry out?

**B. STRATEGIES AND PROGRAMS TO REDUCE FGM**

Now I would like to discuss strategies or programs in place to reduce FGM in your prefecture/region/hospital/health centre.

1. Are you familiar with strategies or programs put in place to reduce FGM?
2. Are/were you involved **in the conceptualization and design** of any of these strategies or programs?
3. Are/were you involved **in the implementation** of any of these strategies or programs?
4. In your opinion are these strategies effective in reducing the practice of FGM within the communities? (If yes, please explain why) (If no, please explain why not).
5. What is the role of religion in these strategies?
6. In your opinion, which actions are effective to reduce the demand in FGM within the community?

**C. FGM PERFORMED BY HEALTH CARE PROVIDERS**

1. We understand that health care providers are increasingly practicing FGM. What do you know about this phenomenon?
2. What do you think about FGM being carried out by health workers?
3. In your opinion what pushes **providers** to practice FGM? What are their motivations?
4. In your opinion, what pushes **families** to ask providers to cut their daughters?
5. Do any of the prevention strategies/programs to reduce FGM target medical personnel who carry out the practice (medicalization of FGM)?
   1. If yes, in your opinion are these strategies effective in ensuring that providers do not carry out FGM? According to you what could be improved?
   2. If no, do you know why they have not been put in place? (Barriers).
   3. Do you think these strategies/programs should be put in place?

**D. TRAINING OF PROVIDERS ON FGM**

Now, I would like to ask you a few questions about the teaching programs on FGM aimed at health care providers that have been implemented in Guinea.

1. Are you aware of teaching programs on FGM aimed at medical, nursing or midwifery students? (Interviewer should indicate the type of provider)
2. If yes, do you know if these teaching programs have been officially evaluated?
   1. If yes, based on these evaluations:
      1. Are these programs correctly implemented?
      2. Is the content appropriate?
      3. Is the content and format acceptable to providers?
3. If no, how feasible would it be to implement these programs?
4. Based **on your perceptions**:
   1. Are these training programs correctly implemented?
   2. Is the content appropriate?
   3. Is the content and format acceptable to providers?
5. What potential challenges would you expect with the implementation of these programs?
6. Finally, what other strategies/programs would you suggest putting in place to reduce FGM by medical personnel (e.g., training, communication strategies (describe content and channels), follow up, evaluation)?
